# Supplementary material for: Distinct neurocognitive bases for social trait judgments of faces in autism spectrum disorder
Source: Transl Psychiatry. 2022 Mar 15;12:104. doi: 10.1038/s41398-022-01870-9 (PMC8924227; doi:10.1038/s41398-022-01870-9)
Supplement: Supplementary file 1 — Supplementary Materials [file 41398_2022_1870_MOESM1_ESM.docx]

*Supplementary Materials for*

**Distinct neurocognitive bases for social trait judgments of faces in**

**autism spectrum disorder**

Hongbo Yu^1^†, Runnan Cao^2^†, Chujun Lin^3^, Shuo Wang^2,4^

^1^ Department of Psychological & Brain Sciences, University of California Santa Barbara, Santa Barbara, CA 93106, USA

^2^ Lane Department of Computer Science and Electrical Engineering, West Virginia University, Morgantown, WV 26506, USA

^3^ Department of Psychological and Brain Sciences, Dartmouth College, Hanover, NH 03755, USA

^4^ Department of Radiology, Washington University in St. Louis, St. Louis, MO 63110, USA

† These authors contributed equally to this work.

Correspondence:

Hongbo Yu (hongbo.yu@psych.ucsb.edu)

Shuo Wang (shuowang@wustl.edu)

**Supplementary Methods**

**Single-neuron recordings and neuronal response to faces.** We recorded from implanted depth electrodes in the amygdala and hippocampus from patients with pharmacologically intractable epilepsy. Target locations in the amygdala and hippocampus were verified using post-implantation CT. At each site, we recorded from eight 40 μm microwires inserted into a clinical electrode as described previously (Rutishauser et al., 2013; Rutishauser, Mamelak, et al., 2006). Efforts were always made to avoid passing the electrode through a sulcus, and its attendant sulcal blood vessels, and thus the location varied but was always well within the body of the targeted area. Microwires projected medially out at the end of the depth electrode and examination of the microwires after removal suggests a spread of about 20-30 degrees. The amygdala electrodes were likely sampling neurons in the mid-medial part of the amygdala and the most likely microwire location is the basomedial nucleus or possibly the deepest part of the basolateral nucleus. Bipolar wide-band recordings (0.1-9000 Hz), using one of the eight microwires as the reference, were sampled at 32 kHz and stored continuously for off-line analysis with a Neuralynx system. The raw signal was filtered with a zero-phase lag 300-3000 Hz bandpass filter and spikes were carefully sorted using a semi-automatic template matching algorithm as described previously (Rutishauser, Schuman, et al., 2006).

We used a 1-back task to acquire neural responses to the same CelebA stimuli from neurosurgical patients. In each trial, a single face was presented at the center of the screen for a fixed duration of 1 second, with uniformly jittered inter-stimulus-interval (ISI) of 0.5-0.75 seconds. Each image subtended a visual angle of approximately 10º. A simple 1-back task required patients to press a button if the present face image was *identical* to the immediately previous image. Nine percent of the trials were one-back repetitions. Each face was shown once unless repeated in one-back trials; and the faces shown in one-back trials were randomly selected for each patient. We excluded responses from one-back trials to have an equal number of responses for each face. This task kept patients attending to the faces, but avoided potential biases from focusing on any particular facial feature (e.g., the color of their eyes or whether they are smiling) or social judgment (e.g., whether they seem happy). The order of faces was randomized for each patient. Stimuli were presented using MATLAB with the Psychtoolbox 3 (Brainard 1997) (<http://psychtoolbox.org>) (screen resolution: 1600 × 1280).

Only units with an average firing rate of at least 0.15 Hz during the entire task were considered. Only single units were considered. Trials were aligned to stimulus onset. We used the mean firing rate in a time window 250 ms to 1250 ms after stimulus onset as the response to each face. Firing rate was then normalized by dividing the mean activity in the baseline (−250 ms to 0 ms relative to stimulus onset). Such normalization was applied in previous studies that analyzed the similarity between single-neuron responses to visual categories (Reber et al., 2019).

**Supplementary Results**

**Neurophysiological results**. For completeness, here we reported the results of the analysis of the neurophysiological data with regard to all 10 social traits. We constructed a social dissimilarity matrix (DM) between identity pairs by computing covariance on the traits between each pair of face identities for control (**Fig. S2a**) and ASD group (**Fig. S2b**), and the neural DM by calculating the covariance across neurons (**Fig. S2c**). We used a bootstrap with 1000 runs to estimate the distribution of DM correspondence for each participant group. In each run, 70% of the data were randomly selected from each participant group and we calculated the correspondence (Spearman’s ρ) between the social trait DM and the neural response DM for each participant group. We then created a distribution of DM correspondence for each participant group. Similar to the analysis of single traits above, we used a permutation test to determine whether there was a significant difference between the correspondence of neural and social DMs in participants with ASD and controls.

We found that the observed correspondence difference between the ASD and control groups is significantly larger than when the difference was estimated with label-shuffled data (permutation *p* < 0.001). In addition, we also used a bootstrapping approach to estimate the distribution of DM correspondence for each participant group and we found that the two distributions were largely separated (**Fig. S2d**; the mean of the ASD distribution was significantly outside the control distribution [*p* < 0.011] and the mean of the control distribution was also significantly outside the ASD distribution [*p* < 0.017]).

**Regression analysis with recognized and unrecognized faces**. We separated the faces into two categories based on whether participants indicated that they recognized the identity of a face. As reported in the main text, we ran two linear regression models to examine whether the two groups exhibited differential association patterns between personality dimensions, and trustworthiness and warmth judgments, separately for recognized and unrecognized faces.

For the regression model with trustworthiness rating, we found that the interaction between group and Factor 3 score was significant for the recognized faces (*B*±s.e.m. = 0.22±0.09, *t* = 2.30, *p* = 0.02, CI = [0.03, 0.40]), but not the unrecognized faces (0.11±0.08, *t* = 1.30, *p* = 0.19, CI = [-0.05, 0.26]). The other group by factor interactions were not significant for the recognized or the unrecognized faces. For the regression model with warmth rating, the interaction between group and Factor 4 score was significant for the recognized faces (0.19±0.09, *t* = 2.08, *p* = 0.04, CI = [0.01, 0.37]) but not for the unrecognized faces (0.07±0.07, *t* = 0.98, *p* = 0.34, CI = [-0.07, 0.21]). The other group by factor interactions were not significant for the recognized or the unrecognized faces.

**Similarity analysis for recognized and unrecognized faces.**As we did for the regression analysis above, we repeated the similarity analysis separately for recognized and unrecognized faces. The same patterns of results as we reported in the main text were obtained when analyzing the recognized (Fig. S4) and unrecognized (Fig. S5) faces separately, demonstrating the robustness of the results.

**Supplementary Figures**

**
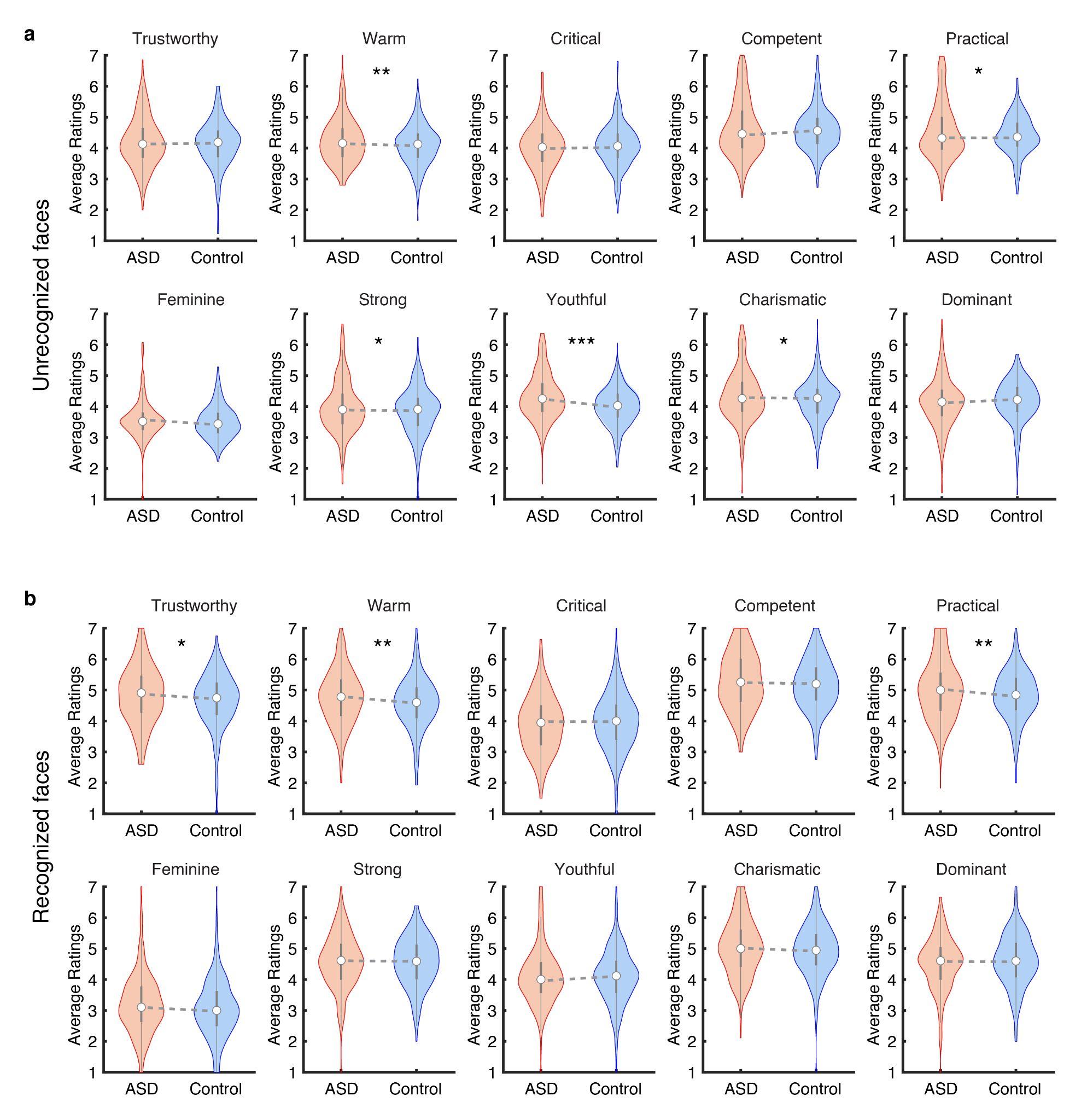
**

**Figure S1.** Results of the face judgment task when separately analyzing the unrecognized (a) and recognized (b) faces. * *p* < 0.05, ** *p* < 0.01, and *** *p* < 0.001.


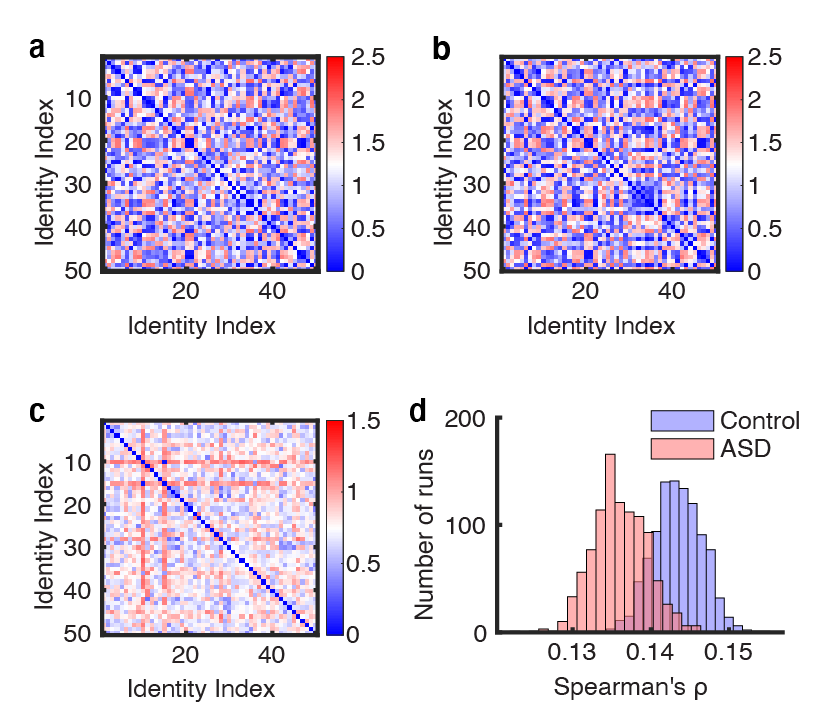


**Figure S2. Results of the analysis of the neurophysiological data with regard to all ten social traits. (a, b)** Average social DM constructed across 10 social traits for control and ASD using consensus ratings acquired from each run of the bootstrapping. **(c)** Neuronal DM constructed across neurons using the average response of each identity. **(d)** Bootstrap distribution of DM correspondence for each participant group. Blue: online controls. Red: online participants self-identified as ASD. Participants with ASD showed a weaker correspondence with the neural response DM compared to controls.


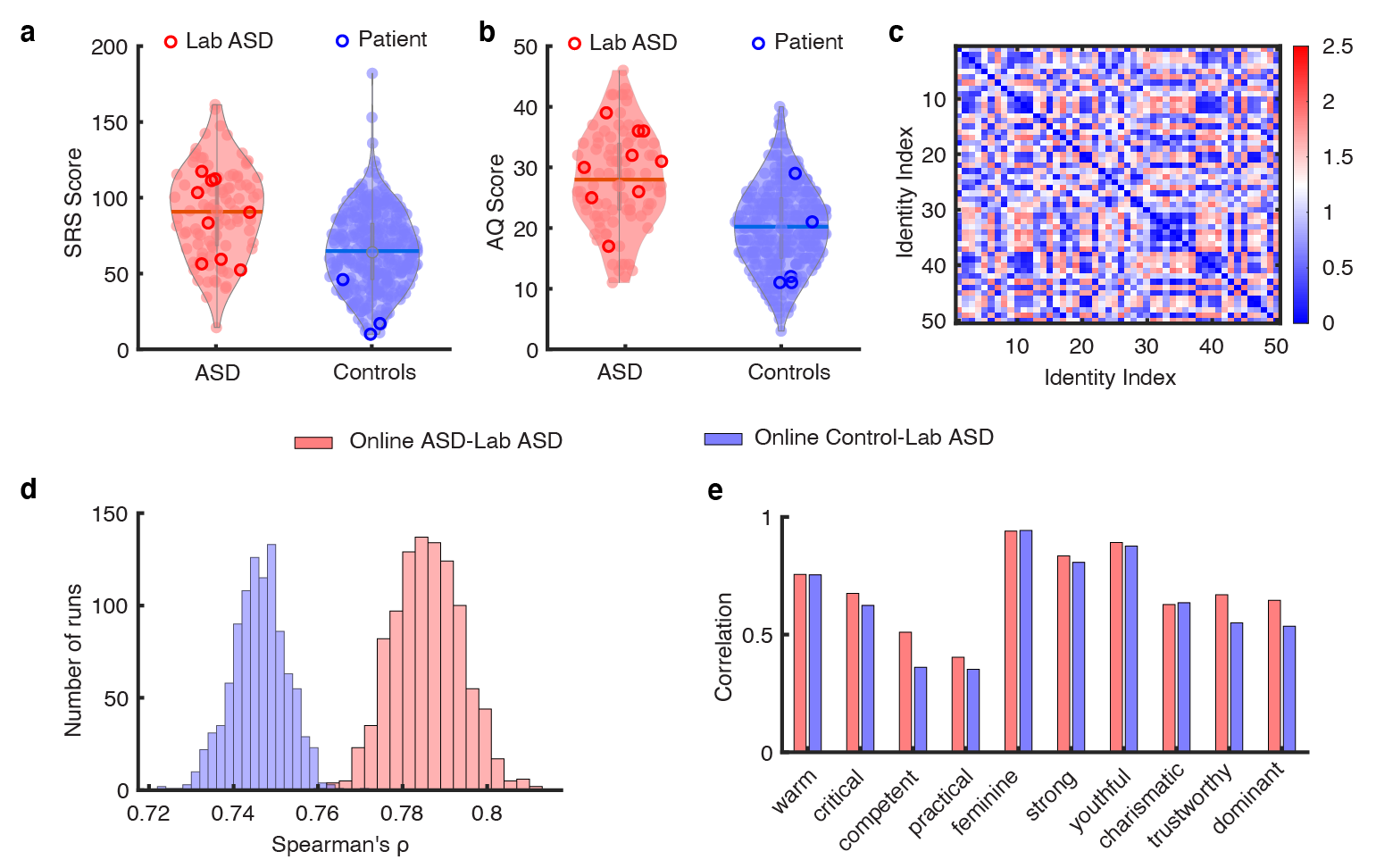


**Figure S3. Comparable autistic scores and face judgments between participants with crowd-source ASD and in-lab participants with diagnosed ASD. (a, b)** The violin plots illustrate the distribution of SRS scores **(a)** and AQ scores **(b)** for online participants self-identified with ASD and online control participants. The dark red circles indicate the SRS and AQ scores from in-lab participants with diagnosed ASD. The dark blue circles indicate scores of intracranial (neurotypical) patients. **(c)** Social trait DM constructed across 10 traits of ratings from in-lab participants with ASD. The social trait DM shows dissimilarity between all pairs of face identities and reflects an overall structure of the social trait judgment space. **(d)** Bootstrap distribution of DM correspondence between in-lab participants with crowd-source participants with ASD (red) and controls (blue). **(e)** Face judgments are highly correlated between in-lab participants with ASD (red) and online crowd-source participants with ASD (blue).


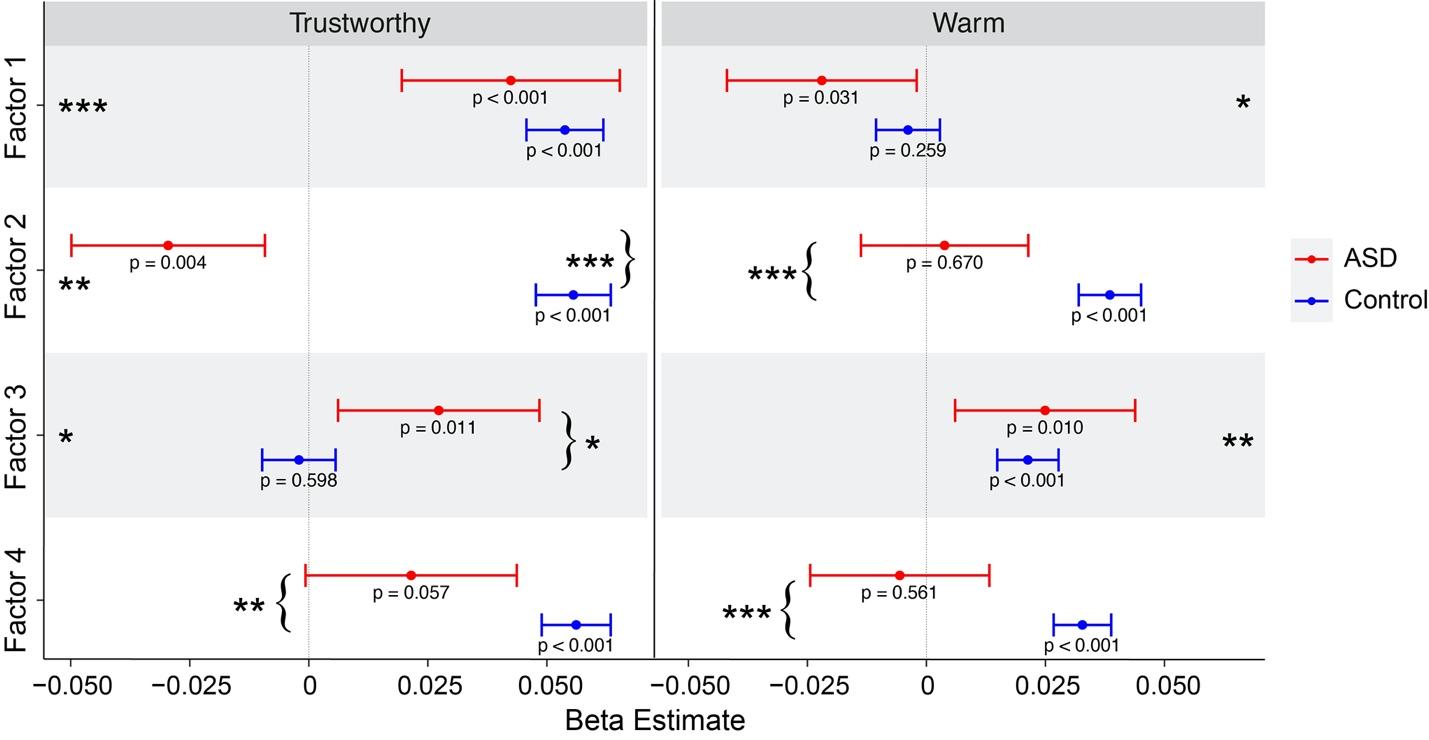


**Figure S4.** **Results of the similarity analysis on recognized faces.** The figure illustrates regression coefficients of each personality dimension (factor) for Trustworthy (left) and Warm (right) judgments. The asterisks on the margins indicate significant main effect of a personality dimension in predicting the social trait judgments, while the asterisks with curly brackets indicate significant group by factor interaction, or in other words, significant group difference in the predictive power of a given personality dimension.


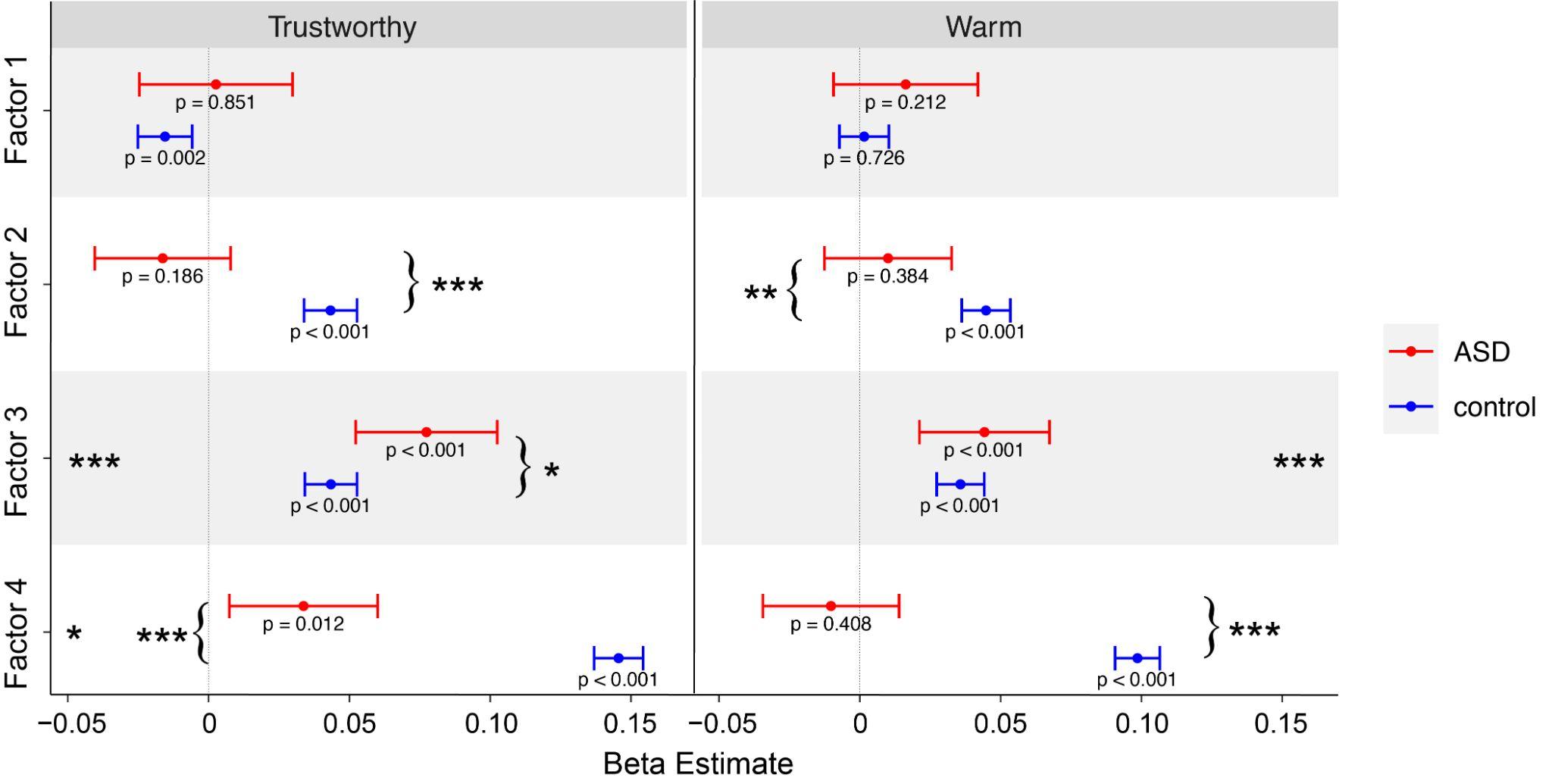


**Figure S5.** **Results of the similarity analysis on unrecognized faces.** The figure illustrates regression coefficients of each personality dimension (factor) for Trustworthy (left) and Warm (right) judgments. The asterisks on the margins indicate significant main effect of a personality dimension in predicting the social trait judgments, while the asterisks with curly brackets indicate significant group by factor interaction, or in other words, significant group difference in the predictive power of a given personality dimension.

**Supplementary Tables**

**Table S1. Top loading items on Factor 1 (‘Autistic traits and social avoidance’): full sample**

| **Questionnaire subscale** | **Loading** |
| --- | --- |
| Social Anxiety: Interaction anxiousness | 0.842 |
| SRS | 0.822 |
| AQ | 0.782 |
| Big 5: Neuroticism | 0.690 |
| Social Anxiety: Audience anxiousness | 0.637 |
| Alexithymia: Difficulty describing emotion | 0.622 |
| Alexithymia: Difficulty identifying emotion | 0.621 |
| Moral Scrupulosity | 0.577 |
| QCAE: Emotion contagion | 0.429 |
| Big 5: Extraversion | 0.601 |
| Apathy: Behavioural motivation | 0.551 |
| Apathy: Social motivation | 0.540 |
| QCAE: Perspective taking | 0.531 |

Note: Summary of item loadings onto the ‘Autistic traits and social avoidance’ factor. The top loading subscales from each questionnaire (|loading| > 0.35) are displayed in descending order. Items with red loadings numbers are reverse-coded. SRS = Social Responsiveness Scale, AQ = Autism-spectrum Quotient (AQ), QCAE = Questionnaire of Cognitive and Affective Empathy.

**Table S2. Top loading items on Factor 2 (‘Empathy and Prosociality’): full sample**

| **Questionnaire subscale** | **Loading** |
| --- | --- |
| QCAE: Proximal responsivity | 0.743 |
| Proscialness | 0.702 |
| Apathy: Emotional sensitivity | 0.633 |
| QCAE: Online simulation | 0.596 |
| QCAE: Peripheral responsivity | 0.594 |
| QCAE: Perspective taking | 0.553 |
| QCAE: Emotion contagion | 0.493 |
| Apathy: Social motivation | 0.402 |
| Big 5: Openness | 0.371 |
| Alexithymia: Externally oriented | 0.512 |
| Dark Factor: Crudelia | 0.551 |
| Dark Factor: Self-centeredness | 0.385 |
| Dark Factor: Moral Disengagement | 0.380 |

Note: Summary of item loadings onto the ‘Empathy and Prosociality’ factor. The top loading subscales from each questionnaire (|loading| > 0.35) are displayed in descending order. Items with red loadings numbers are reverse-coded. QCAE = Questionnaire of Cognitive and Affective Empathy.

**Table S3. Top loading items on Factor 3 (‘Antisociality’): full sample**

| **Questionnaire subscale** | **Loading** |
| --- | --- |
| Dark Factor: Machiavellianism | 0.671 |
| Dark Factor: Frustralia | 0.643 |
| Dark Factor: Crudelia | 0.606 |
| Dark Factor: Spitefulness | 0.575 |
| Dark Factor: Moral Disengagement | 0.496 |
| Dark Factor: Egoism | 0.482 |
| Dark Factor: Psychopathy | 0.477 |
| OUS: Instrumental harm | 0.472 |
| Dark Factor: Self-centeredness | 0.409 |
| Alexithymia: Difficulty identifying emotion | 0.404 |
| Moral scrupulosity | 0.377 |
| Big 5: Conscientiousness | 0.406 |

Note: Summary of item loadings onto the ‘Antisociality’ factor. The top loading subscales from each questionnaire (|loading| > 0.35) are displayed in descending order. Items with red loadings numbers are reverse-coded. OUS = Oxford Utilitarianism Scale

**Table S4. Top loading items on Factor 4 (‘Social agreeableness’): full sample**

| **Questionnaire subscale** | **Loading** |
| --- | --- |
| Big 5: Agreeableness | 0.552 |
| OUS: Impartial beneficence | 0.537 |
| Social support | 0.436 |
| Prosocialness | 0.429 |
| QCAE: Proximal responsivity | 0.386 |
| Apathy: Emotional sensitivity | 0.383 |
| Dark Factor: Self-centeredness | 0.404 |
| Dark Factor: Machiavellianism | 0.363 |

Note: Summary of item loadings onto the ‘Social agreeableness’ factor. The top loading subscales from each questionnaire (|loading| > 0.35) are displayed in descending order. Items with red loadings numbers are reverse-coded. OUS = Oxford Utilitarianism Scale, QCAE = Questionnaire of Cognitive and Affective Empathy.

**Table S5. Top loading items on Factor 1 (‘Autistic traits and social avoidance’): sex ratio matched sample**

| **Questionnaire subscale** | **Loading** |
| --- | --- |
| Social Anxiety: Interaction anxiousness | 0.814 |
| SRS | 0.710 |
| AQ | 0.691 |
| Big 5: Neuroticism | 0.671 |
| Social Anxiety: Audience anxiousness | 0.620 |
| Alexithymia: Difficulty describing emotion | 0.618 |
| Alexithymia: Difficulty identifying emotion | 0.599 |
| Moral Scrupulosity | 0.599 |
| QCAE: Emotion contagion | 0.589 |
| Apathy: Emotional sensitivity | 0.417 |
| Big 5: Extraversion | 0.485 |
| Apathy: Behavioral activation | 0.409 |
| Dark Factor: Self-centeredness | 0.408 |

Note: Summary of item loadings onto the ‘Autistic traits and social avoidance’ factor. The top loading subscales from each questionnaire (|loading| > 0.35) are displayed in descending order. Items with red loadings numbers are reverse-coded. SRS = Social Responsiveness Scale, AQ = Autism-spectrum Quotient (AQ), QCAE = Questionnaire of Cognitive and Affective Empathy.

**Table S6. Top loading items on Factor 2 (‘Empathy and Prosociality’): sex ratio matched sample**

| **Questionnaire subscale** | **Loading** |
| --- | --- |
| QCAE: Proximal responsivity | 0.666 |
| QCAE: Perspective taking | 0.621 |
| Proscialness | 0.615 |
| QCAE: Peripheral responsivity | 0.611 |
| Apathy: Emotional sensitivity | 0.545 |
| Apathy: Social motivation | 0.525 |
| QCAE: Online simulation | 0.502 |
| QCAE: Emotion contagion | 0.429 |
| Big 5: Openness | 0.393 |
| Big 5: Extraversion | 0.369 |
| Alexithymia: Externally oriented | 0.447 |

Note: Summary of item loadings onto the ‘Empathy and Prosociality’ factor. The top loading subscales from each questionnaire (|loading| > 0.35) are displayed in descending order. Items with red loadings numbers are reverse-coded. QCAE = Questionnaire of Cognitive and Affective Empathy.

**Table S7. Top loading items on Factor 3 (‘Antisociality’): sex ratio matched sample**

| **Questionnaire subscale** | **Loading** |
| --- | --- |
| Dark Factor: Frustralia | 0.644 |
| Dark Factor: Machiavellianism | 0.618 |
| Dark Factor: Crudelia | 0.577 |
| Dark Factor: Spitefulness | 0.540 |
| Alexithymia: Difficulty identifying emotion | 0.528 |
| Scrupulosity | 0.507 |
| Dark Factor: Egoism | 0.480 |
| OUS: Instrumental harm | 0.454 |
| Dark Factor: Psychopathy | 0.450 |
| Dark Factor: Moral Disengagement | 0.438 |
| Big 5: Conscientiousness | 0.406 |

Note: Summary of item loadings onto the ‘Antisociality’ factor. The top loading subscales from each questionnaire (|loading| > 0.35) are displayed in descending order. Items with red loadings numbers are reverse-coded.

**Table S8. Top loading items on Factor 4 (‘Social agreeableness’): sex ratio matched sample**

| **Questionnaire subscale** | **Loading** |
| --- | --- |
| Big 5: Agreeableness | 0.567 |
| Apathy: Behavioural activation | 0.542 |
| OUS: Impartial beneficence | 0.461 |
| Social support | 0.412 |
| Big 5: Conscientiousness | 0.389 |
| Prosocialness | 0.381 |

Note: Summary of item loadings onto the ‘Social agreeableness factor. The top loading subscales from each questionnaire (|loading| > 0.35) are displayed in descending order. OUS = Oxford Utilitarianism Scale.
